# Superheated liquid and supercritical denatured ethanol extraction of antioxidants from Crimson red grape stems

**DOI:** 10.1002/fsn3.246

**Published:** 2015-05-22

**Authors:** Jonathan Wenzel, Cheryl Storer Samaniego, Lihua Wang, La'Shyla Nelson, Korrine Ketchum, Michelle Ammerman, Ali Zand

**Affiliations:** ^1^ Kettering University1700 University AveFlintMichigan48504

**Keywords:** Antioxidant activity, Crimson seedless grape, FRAP assay, grape stem extracts, supercritical ethanol extraction, superheated liquid ethanol extraction, TPC

## Abstract

Grapes are widely known for health benefits due to their antioxidant content. In wine production, grape stems are often discarded, though they has a higher content of antioxidants than the juice. The effectiveness of using an environmentally friendly solvent, ethanol, as a superheated liquid and supercritical fluid to extract antioxidant compounds from grape stems of organically grown Crimson Seedless grapes was evaluated. The Ferric Reducing Ability of Plasma (FRAP) assay and the Total Phenolic Content (TPC), or Folin‐Ciocalteu assay, were used to quantify the antioxidant power of grape stem extracts. The extractions were performed at temperatures between 160°C and 300°C at constant density. It was found that the optimal extraction temperature was 204°C, at superheated liquid conditions, with a FRAP value of 0.670 mmol Trolox Equivalent/g of dry grape stem. The FRAP values were higher than other studies that extracted antioxidants from grape stems using single‐pass batch extraction.

## Introduction

Grapes are the most commonly grown fruit in the world with over 67.1 million tons grown in 2010 (Ghafoor et al. 2012). Eighty percent of grape crops are used in the production of wine, resulting in the production of nearly 13 million tons of grape pomace (Spatafora et al. 2013). Grape pomace, consisting of seeds, peels, and stems, is often discarded as waste. However, disposal of grape pomace may present environmental concerns due to its polyphenolic content and related phytotoxicity during composting as well as an increased biochemical oxygen demand.(Aliakbarian et al. 2012) During grape vinification, stems are separated and discarded due to impact upon the flavor of juice and wine.

Since antiquity grapes and wine have been prized for health benefits and promoting longevity. Antioxidants inhibit oxidation free‐radical production leading to cell damage and death. However, like many fruits, the highest concentration of antioxidants in grapes does not occur in the pulp, the origin of most of the antioxidant compounds of wines and juices. For example, seeds of the red rose grape have 100 times the antioxidant power and the peel contains 20 times the antioxidant power compared to pulp (Guo et al. 2003). Though not as frequently studied, grape stems also contain phenolic compounds and exhibit similar antioxidant power as grape seeds (Souquet et al. 2000; Anastasiadi et al. 2009). In addition, grape stem antioxidant activity is constant through growth and maturation of the grape in comparison to the seeds, leaves, and peels (Doshi et al. 2006). As a consequence, grape stems present stable economic potential since they exhibit antioxidant power and are ordinarily discarded during processing.

Red wine consumption is associated with decreased cardiovascular incidences (Renaud et al. 2004). Many health benefits of wine and grapes come from phenolic compounds such as flavonols, procyanidins, and phenolic acids, including gallic acid, catechin, quercetin, resveratrol, and viniferin which exhibit antioxidant, antiallergenic, anti‐mutagenic, antimicrobial, anti‐carcinogenic, anti‐fungal, and anti‐inflammatory properties (Han 2007; Katalinic et al. 2010; Oliveira et al. 2013). Increased levels of phenolic compounds are potentially produced in grapes when stressed by fungal attack or for free‐radical neutralization when exposed to ultraviolet light (Douillet‐Breuil et al. 1999; Threlfall et al. 1999). In addition to medicinal uses, grape extracts' oxidative inhibition characteristics are beneficial in cosmetics as they bind collagen in the skin, promoting a youthful appearance (Peralbo‐Molina et al. 2013). Proanthocyanidins protect the body from sun damage, enhance vision, promote flexibility in joints, arteries and cardiac tissue, and improve blood circulation.(Peralbo‐Molina et al. 2013) As a consequence of their high phenolic content, grape stems and pomace present an opportunity for pharmaceutical and herbal supplements.

Several methods have been evaluated for extracting the antioxidant, phenolic compounds from grape residues such as liquid–solid extraction and supercritical fluids, all of which use a variety of solvents (Louli et al. 2004; Pinelo et al. 2007; Gonzalez‐Centeno et al. 2012). For pharmaceutical, cosmetic, and food industries, it is important that the solvents are relatively nontoxic, inexpensive, and that methods are selective and efficient in extracting antioxidant compounds. In addition, solvents that are environmentally benign and naturally occurring are attractive, narrowing the list of potential solvents. For selectivity, the polarity of the phenolic compounds necessitates a slightly polar solvent. Extraction efficiency is dictated by solvent selection as well as operating parameters such as temperature, density, acidity, and hold time.

Several extraction solvents have been evaluated and compared for extracting phenolic compounds from grape pomace, seeds, and to lesser extent, stems using both batch or flow‐through methods. For conventional liquid‐solid extraction and soxhlet extraction, ethyl acetate, methanol, ethanol, potassium hydroxide‐water, water, and mixtures have been evaluated (Louli et al. 2004; de Campos et al. 2008; Oliveira et al. 2013). Also water and alcohols have been acidified to enhance extraction (Rockenbach et al. 2011; Peralbo‐Molina et al. 2013). These solvents may be particularly effective at extracting phenolic and polyphenolic compounds, but are not particularly selective as is the case with ethyl acetate (de Campos et al. 2008) or require long extraction times (Casazza et al. 2012). A potential drawback of soxhlet extraction includes degradation of antioxidant compounds which are sensitive to light and oxygen. Superheated liquid extraction (SHLE) has also been evaluated, in particular water and acidified ethanol and water (Luque‐Rodriguez et al. 2007; Aliakbarian et al. 2012; Peralbo‐Molina et al. 2012). SHLE has advantages, for example, SHLE with water takes advantage of the high dielectric constant of liquid water to extract polar compounds and the higher extraction temperature to maximize yield.

Another means of performing extractions is using supercritical fluids. Carbon dioxide, water, and ethanol are notable solvents for supercritical fluid extraction in terms of excellent solvating capabilities and are generally recognized as safe by the United States Food and Drug Administration. Supercritical carbon dioxide and liquid carbon dioxide are extensively used as an environmentally friendly, nontoxic solvent to extract caffeine from coffee beans and tea, hops extraction, and extraction of flavors and essences (DeSimone 2002). At moderate pressures, supercritical carbon dioxide is relatively nonpolar (Leeke et al. 2005). Consequently, slightly polar antioxidant compounds are not particularly soluble in supercritical carbon dioxide near its critical pressure. Since the dielectric constant of carbon dioxide increases with increasing pressure, extraction of antioxidants necessitates pressures greater than 300 bar, which is not particularly selective for grape pomace extraction (Keyes and Kirkwood 1930; Murga et al. 2000). To facilitate extraction of phenolic compounds with supercritical carbon dioxide, ethanol is commonly added as a modifier to increase the polarity of the mixture which can be effective for extracting antioxidants from grape pomace (Casas et al. 2010; Oliveira et al. 2013). This decreases the extraction pressure, but increases the extraction temperature of carbon dioxide.

The aim of this study was to evaluate the effects of using superheated liquid ethanol and supercritical ethanol for extracting antioxidants from stems of seedless red grapes. The effect of extraction temperature upon antioxidant potential and total phenolic content, using this solvent was determined and compared against other published extraction techniques. Superheated liquid or supercritical ethanol extraction is advantageous as a solvent because ethanol is generally recognized as safe in the processing of foods and drugs, it has a higher dielectric constant than carbon dioxide, it does not involve the use of a gas, and the higher temperature may result in more efficient extraction of antioxidant compounds.

## Materials and Methods

### Chemicals

The following reagent grade or greater chemicals were used for the FRAP assay and the TPC assay: concentrated hydrochloric acid, ferric chloride hexahydrate, HPLC grade ethanol, and 2,4,6‐Tris(2‐pyridyl)‐s‐triazine (TPTZ) from Sigma Aldrich; ACS certified grade Ferrous sulfate heptahydrate and glacial acetic acid from Fisher Scientific; Sodium acetate trihydrate (≥99%) from Avantor; Gallic acid monohydrate from Acros Organics; Folin‐Ciocalteu reagent from Merck; Sodium carbonate monohydrate from J. T. Baker Chemical and Ultrapure water from Cayman Chemical Company. Ethanol that was used for the extraction was 95% pure and denatured with 5% wood spirit. Nitrogen (99.998%) and helium (99.999%) were from Praxair. Chemicals were used without further purification. All chemicals originated from the United States.

### Plant material

Crimson Seedless organic table grapes evaluated were grown in California in the 2013 growing season. The Crimson Seedless grape is a late‐harvest table grape commonly found in local grocers in the United States. After the grapes were removed from the stems, the stems and branches were dried in an oven at 55°C for 90 to 120 min. The stems were then separated from the branches and stored in vials in cool, dark air tight containers until extraction. Stems were extracted whole or were ground using a coffee grinder prior to extraction. The particle sizes of the ground stems ranged from 20 to 50 mesh.

### Extraction system

**Figure 1:**
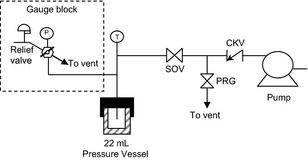
Process flow diagram for the batch extraction system.

Extractions were performed in batches in a custom‐built extraction system, Figure 1, with a total volume of 24 mL. The major components are a heated pressure vessel, pressure gauge block with relief valve, thermocouple with indicator, and a pump and purge section. All wetted parts were 316 stainless steel. The 22 mL bolt‐closure pressure vessel was a from Parr Instruments Company (Moline, Illinois) model 4742, with a single port and graphite gasket with a maximum allowable working pressure of 587 bar at 350°C. The pressure gage was an Ashcroft 3 ½” diameter Bourdon tube pressure gage with a range of 0 to 5000 psig (0 to 346 bar) and reading increments of 50 psi (3.4 bar). The pump was an Eldex Laboratories Optos Series model 2SM metering piston pump. The shut off valve (SOV) and purge valve (PRG) are two‐way straight valves with a regulated stem, Autoclave Engineers part number SW4081. The poppet‐style check valve (CKV) prevented backflow to the pump, Swagelok, SS‐CHS4‐20. Tubing used was a 6.35‐mm outer diameter seamless tubing. The pressure vessel was heated using a heating mantle and temperature was controlled manually with a variac.

### Extraction method

Prior to use, the extraction system was scoured, pressurized with acetone, then purged, and dried with nitrogen. For each experiment, ethanol was purged of oxygen by bubbling nitrogen gas for 15 min. Approximately 0.50 grams of dried grape stems, whole or ground, and 14.6 g of ethanol for a constant density of 0.61 g/mL, were placed in the pressure vessel. The pressure vessel was then purged with nitrogen, sealed, and connected to the batch extraction system. It was heated to the target temperature and the temperature was held for the required hold time. Next the pressure vessel was allowed to cool, opened, and the liquid extract suctioned out using a transfer pipette. Without post‐treatment, the liquid extract was stored in a glass vial blanketed with nitrogen, double‐sealed, and stored in the dark at 4°C to protect the sample from decomposition. The extract was analyzed using the FRAP and TPC assays.

### Antioxidant activity: FRAP assay

The Ferric Reducing Ability of Plasma (FRAP) assay was performed as previously described, (Benzie and Strain 1996) where the absorbance of the analytes were measured at 590 nm with a Cary 300 Bio UV/Vis Spectrophotometer. The FRAP reagent was prepared daily with a 0.3 M acetate buffer, 10 mM TPTZ in 40 mM HCl, and 200 mM FeCl_3_ and maintained at 37°C once prepared. A calibration curve for Fe^2+^ was generated by using FeSO_4_ solutions with FRAP reagent and compared with a blank solution of FRAP reagent. Ferrous sulfate gives a change in absorbance that is one half of that given by an equivalent Trolox molar concentration.(Benzie and Strain 1996). Extract samples were assessed by mixing 900 *μ*L FRAP reagent with 0.5 to 15 *μ*L sample and (120 *μ*L – sample volume) of ultrapure water. Sample readings were assessed at 10 min in replicate and averaged with standard deviation. Average sample absorbance values were subtracted by the absorbance due to the color of the sample to normalize the data. Corresponding Fe^2+^ concentrations generated by the samples were determined by the calibration curve and converted to mmol Trolox equivalent/gram of dry grape stems (mmol TE/g).

### Total phenolic content

The Total Phenolic Content (TPC) or Folin–Ciocalteu (F‐C) assay, determines the oxidation of phenolic compounds by a molybdotungstate reagent yielding a colored product with *λ*_max_ at 745–750 nm. (Folin and Ciocalteu 1927; Singleton and Rossi 1965) Since phenolic compounds are the primary antioxidants in plants, the extracts were also analyzed by the TPC assay. The TPC assays of the extracts were carried out using a previously described procedure utilizing a 96‐well microplate.(Ainsworth and Gillespie 2007) Ethanol solutions of gallic acid (50 *μ*mol/L to 1000 *μ*mol/L) were used as the standards to create the calibration curve. The samples were diluted 1:20 in ethanol. The absorbance was measured using a BioTek Synergy HT microplate reader. Total phenolic content was determined as milligrams of gallic acid equivalent/g of dry grape stem (mg GAE/g). The TPC assay was performed in duplicate on different days.

### Statistical analysis

Data were evaluated by ANOVA tests at a 0.05 level of significance. The effects of varying grape stem morphology, temperature, and hold time were evaluated. At least four FRAP analyses and four TPC analyses were performed for each extraction sample. All experimental results were reported as mean values with corresponding standard deviations. A *P*‐value less than 0.05 was considered statistically significant. The relationship between FRAP and TPC analysis was described by the correlation coefficient, *R* ^2^.

## Results and Discussion

The antioxidant ability of superheated liquid and supercritical ethanol extraction of dried grape stems was characterized for effects of temperature, hold time, and effects of morphology by comparing ground and whole grape stems. The extraction of dried grape stems was evaluated at a constant directly measured ethanol density of 0.61 g/mL in the pressure vessel from 160°C to 300°C. The critical point of pure ethanol is 241°C at 63 bar of pressure, given the similarity in critical points of ethanol and methanol, the critical point of denatured ethanol will be within 2% of pure ethanol. The density of ethanol was chosen in order to ensure that it is a superheated liquid or a supercritical fluid, depending on temperature. Ground grape stems were used to evaluate the effects of temperature. The extraction of whole versus ground grape stems was compared at hold times from 0 to 90 min in increments of 30 min at 300°C and 111 ± 5 bar. No significant difference was observed by varying hold time between whole and ground grape stems; consequentially, ground grape stems were used for subsequent experiments.

### Effect of temperature upon FRAP antioxidant potential

**Figure 2:**
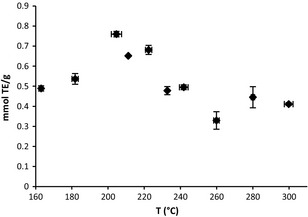
Effect of temperature upon antioxidant activity of ground grape stem extracts as measured by the FRAP assay, 0.5 g grape stem, ethanol density 0.61 g/mL, and hold time of 60 min.

The effect of extraction temperature upon the antioxidant activity as measured by the FRAP assay of ground grape stem extracts was evaluated with a hold time of 60 min, Figure 2. The effect of temperature was statistically significant with *P* ≤ 0.05. At 163.0 ± 0.9°C, the FRAP value was 0.488 ± 0.012 mmol TE/g. As temperature increased to 204.6 ± 2.8°C, the pressure was 34.8 ± 1.2 bar, and the FRAP value increased to a maximum of 0.759 ± 0.015 mmol TE/g, indicating greater antioxidant potential. Above 204°C, the FRAP value declined to 0.476 ± 0.012 mmol TE/g at 241.8 ± 2.4°C, which is near the critical point of ethanol. The minimum FRAP value was 0.329 ± 0.044 mmol TE/g at 259.9 ± 1.5°C; however at 280.9 ± 0.9°C and 299.8 ± 2.8°C, the FRAP values were within experimental error at 0.444 ± 0.053 mmol TE/g and 0.412 ± 0.006 mmol TE/g, respectively. The standard deviation of temperature and pressure reflects the temperature variation during extraction, whereas the standard deviation of the FRAP value comes from multiple FRAP measurements of the same extract. The measured FRAP value of the ethanol used for this study was approximately zero. From a process perspective, these results are informative. Since the density was held constant and the highest FRAP activity was at 204°C, which is below the critical point, the extraction at this condition is at a lower pressure compared with the highest temperature experiment at 300°C. Thus, a less expensive extraction vessel could be used since the pressure rating would be lower. It would require less energy to heat up ethanol and smaller heat exchangers to recover the energy.

Literature providing FRAP activity of grape stem extracts is scarce and there is a lack of references for superheated liquid and supercritical ethanol extraction of grape stems, particularly those of the Crimson Red variety. In comparing other published extraction methods, there is a challenge since there is a lack of uniformity in reporting FRAP values. However, there was some work on acidified superheated liquid extraction, using ethanol–water mixtures of red grape peels. (Luque‐Rodriguez et al. 2007) Extraction of grape stems has been evaluated in limited studies using methanol, methanol/water, acetone/water, and acidified methanol–water mixtures at near‐ambient conditions with temperatures less than 40°C, Table 1. None of these studies systematically evaluated effects of extraction temperature. While it is difficult to draw a direct comparison since the grape stems used in this study were a different species from a different year and country, it is remarkable the extracts produced by this study yielded higher FRAP values in a single‐pass batch extraction which may show that superheated liquid ethanol extraction technique holds potential.

**Table 1:** Comparison of FRAP and TPC values for various methods of extracting grape stems, seeds, and pomace

Investigator	Material	Extraction Method	FRAP	TPC
*This work*	Red crimson grape stems grown in California	Superheated liquid ethanol extraction or supercritical ethanol extraction of dried stems, 160°C < *T* < 300°C, *t* = 1 h	0.329–0.759^a^	35.0–65.2^b^
Anastasiadi et al. (2012)	Red grape stems grown in Greece	Five sequential extractions of dried stems using MeOH/H_2_O/HCl (90:9.5:0.5 v/v) in ultrasonic bath *T* < 35°C	1.5–2.4^c^	5.4–14.3^b^
Gonzalez‐Centeno et al. (2012)	Red grape stems grown in Spain	Sequential extractions of ground stems using eight acetone/water (80:20 v/v) extractions followed by three methanol/water (60:40 v/v) *T* = 40°C, P = 1500 psi, *t* = 4 min	0.26–0.67^a^	47.1–96.4^b^
Balik et al. (2008)	Grape stems grown in Czech Republic	90% methanol, no further information	0.027–0.053^a^	
Llobera and Cañellas (2007)	Red grape stems grown in Spain	Sequential extractions of ground grape stems using methanol/water (50:50 v/v) extraction and a acetone/water (70:30 v/v) extraction *T* = room temp, *t* = 60 min		116^b^
Rockenbach et al. (2011)	Red grape pomace from Brazil	MeOH/HCl (99.9:0.1 v/v), *T* = 4°C, *t* = 1 h	0.11–0.25^a^	32.6–74.7^b^
Katalinic et al. (2010)	Red grape skin grown in Croatia	EtOH/H_2_O (80:20 v/v), *T* = 60°C, *t* = 1 h		0.7–3.5^d^
Maier et al. (2009)	Red grape seeds grown in Germany	Sequential extractions twice with Methanol with 0.1%HCl (v/v) *t* = 120 min		0.11–2.03^b^

a mmol TE/g stem.

b mg GAE/g dry matter.

c mmol TE/g extract.

d mg GAE/g fresh berry.

The experimentally observed trend of FRAP activity with temperature may be due to changes in solvent properties with temperature and decomposition or alteration of the extract. As temperature increases, more material may be extracted due to an increase in diffusivity within the extraction medium, as well as relaxation of the plant matrix, possibly explaining the increase in FRAP activity from 160 to 204°C. However, as the temperature increases in superheated liquid ethanol, the dielectric constant decreases from 8.8 at 160°C to 4.0 at 233°C. The dielectric constant continues to decrease up to ethanol's critical point (Newton et al. 1962). Antioxidant phenolic compounds are slightly polar and are more likely to be solvated in slightly polar fluids. In addition, it is expected that as temperature increases, some of the phenolic antioxidant compounds may decompose (Palma et al. 2001).

The decrease from the maximum FRAP value at 204°C may be due to a decrease in dielectric constant, hence a diminished ability to extract antioxidant compounds as well as the decomposition of the compounds. Similar trends have been noted with the extraction of grape vine shoots as well as red grape peels, using acidified superheated liquid ethanol (Delgado‐Torre et al. 2012; Peralbo‐Molina et al. 2012, 2013). In the acidified superheated liquid ethanol extraction of red grape peels, the highest amount of molecular features was detected at 220°C, the maximum temperature for the study (Peralbo‐Molina et al. 2012). In the acidified superheated liquid ethanol–‐water extraction of grape vine shoots, the amount of hydroxymethylfurfural reached a maximum at 200°C while the total phenolic content reached a maximum at 240°C. Furthermore at temperatures less than 160°C extracts yielded reduced total phenolic concentration (Delgado‐Torre et al. 2012).

While supercritical fluids are notable for their solvating powers due to enhanced diffusion and mass transfer, the FRAP values for supercritical ethanol extraction, *T* > 241°C, were lower than those of superheated ethanol extraction, as seen in Figure 2. Compounds may more readily diffuse through supercritical ethanol, however due to the lower dielectric constant, less antioxidants may be extracted, and when the compounds are extracted, they may decompose. Hence supercritical ethanol may not be a good extraction medium for antioxidant compounds unless it is used as a modifier with carbon dioxide, resulting in a lower temperature and higher dielectric constant.

### Effect of temperature upon total phenolic content

**Figure 3:**
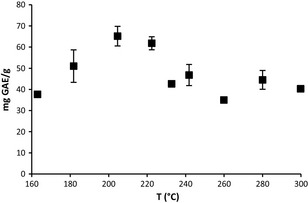
Effect of temperature upon total phenolic content of ground grape stem extracts, 0.5 g grape stem, ethanol density 0.61 g/mL, and hold time of 60 min.

**Figure 4:**
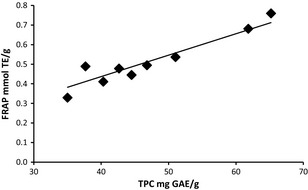
Correlation between TPC and FRAP of ground grape stem extracts (*y* = 0.0109x, *R* ^2 ^= 0.8967), 0.5 g grape stem, ethanol density 0.61 g/mL, and hold time of 60 min.

The effect of temperature on the total phenolic content (TPC) of the grape stem extracts was also evaluated, Figure 3. The TPC of the grape stem extracts as related to temperature followed a trend similar to the antioxidant potential measured by the FRAP assay. At 163.0 ± 0.9°C the TPC value was 37.7 ± 1.1 mg GAE/g. The TPC values increased with increasing temperature reaching a maximum TPC value of 65.2 ± 4.6 mg GAE/g at 204.6 ± 2.8°C. Above this temperature, the TPC value declined to 46.8 ± 5.0 mg GAE/g at 241.8 ± 2.4°C, which is near the critical point of ethanol. The minimum TPC value was 35.0 ± 1.1 mg GAE/g at 259.9 ± 1.5°C. The FRAP and TPC data had a significant correlation with *P* ≤ 0.05, with a coefficient of correlation of 0.9517, and the data was found to be linearly correlated with an *R* ^2 ^= 0.8967, Figure 4.

Other works have presented that FRAP and TPC data are linearly correlated and significant for grape skin (Katalinic et al. 2010) grape seeds (Maier et al. 2009) and grape stems extracted with acidified methanol (Anastasiadi et al. 2012). Literature on TPC for grape stem extracts is scarce. Table 1 provides a comparison of TPC values for grape stem extracts, using different methods and species. None of these studies evaluated the effect of extraction temperature in grape stem extraction upon TPC values or the use of supercritical or superheated liquid ethanol for extraction. However, the TPC values between the different methods were comparable.

## Conclusions

This study clearly demonstrates, for the first time, that superheated liquid ethanol, without acidification or addition of water, may be used to extract antioxidant compounds from grape stems. The grape stem extracts produced had a high antioxidant capacity as measured by the FRAP and TPC assay in comparison to supercritical ethanol extraction and to other extraction methods performed at near ambient conditions. Extraction at superheated liquid ethanol conditions may reach equilibrium quickly and antioxidants can be extracted in high amounts from grape stems. The optimal extraction temperature as measured by the FRAP and TPC assay found in this study was 204°C.

## Conflict of Interest

None declared.
